# Detecting stochastic multiresonance in neural networks via statistical complexity measure

**DOI:** 10.1038/s41598-024-55997-4

**Published:** 2024-03-04

**Authors:** Yazhen Wu, Zhongkui Sun

**Affiliations:** 1 School of Mathematics and Statistics, Northwestern Polytechnical University, Xi’an, 710129 China; 2 Maths and Information Technology School, Yuncheng University, Yuncheng, 044000 China

**Keywords:** Neuroscience, Mathematics and computing

## Abstract

This paper employs statistical complexity measure (SCM) to investigate the occurrence of stochastic multiresonance (SMR) induced by noise and time delay in small-world neural networks coupled with FitzHugh-Nagumo (FHN) neurons. Our findings reveal that SCM exhibits four local maxima at four optimal noise levels, providing evidence for the occurrence of quadruple stochastic resonances. When time delay $\tau$ is taken into account in the information transmission, under moderate noise levels, SCM shows several local maxima when $\tau = nT_{e}$ with $n$ being a positive integer and $T_{e}$ being the period of subthreshold signal. This indicates the appearance of delay-induced SMR at the multiples of the period of subthreshold signal. Intriguingly, at low noise levels, a strong coherence between time delay and neuronal firing dynamics emerges at $\tau = nT_{e} - 2$, as confirmed by a series of SCM maxima at these time delays. Furthermore, the study demonstrates that by adjusting the degrees and sizes of small-world networks, as well as the coupling strength, it is possible to optimize the strength of delay-induced SMR, thus maximizing the detection capability of subthreshold signal. The research results may provide us with an effective approach for understanding the role of time delay in signal detection and information transmission.

## Introduction

It’s well known that noise can lead to astonishingly constructive outcomes in nonlinear dynamical systems. Especially stochastic resonance (SR)^1^, as the most representative example of this fact, is a phenomenon that the response of a nonlinear system to a weak input periodic signal can be amplified by the assistance of optimal noise level. SR has been observed in various types of nonlinear systems, such as physical, chemical, mechanical, biological systems^2–5^. In the context of neural systems, SR has been documented in the caudal photoreceptors of crayfish and cricket^6,7^, predatory behavior of paddlefish^8^ and mammalian brain^9,10^. Numerous experiments and theoretical studies have demonstrated that injecting appropriate noise into sensory neurons can significantly enhance their ability to process weak input signals^11,12^. Consequently, SR is considered as an important tool for detecting weak signals and gaining insights into information transmission within neural networks.

In recent decades, SR has been thoroughly explored in numerous neural network systems. In a comprehensive study by Perc et al*.*, the effects of scale-free network topology on pacemaker-driven SR were thoroughly examined, revealing its remarkable efficacy in facilitating the transmission of weak localized rhythmic activity across the entire network^13^. Kwon et al*.* revealed that spatially correlated noise can significantly enhance the coherence resonance (CR) and reinforce the clustering structure of the network^14^. Furthermore, Liu et al. shed light on the potential of appropriately applied noise and electromagnetic induction in promoting SR and modulating the response of envelope modulation signals within neural networks^15^. Notably, a detail numerical investigation of SMR in a FHN neural network model was reported by Sun et al., who demonstrated that subthreshold signal could be detected successfully at more than one optimal noise level^16^. Together, these findings illustrate the substantial enhancement of information transmission or signal detection capabilities in neural networks through the application of noise-induced SR.

In fact, time delay is unavoidable during the process of information transmission in neural systems because of the limited speed at which action potentials propagate across axons. And it has been discovered that time delay has great influences on dynamical behaviors of coupled neural networks, such as enhancing or decreasing phase synchronization^17^, improving spatial–temporal order^18^, as well as inducing synchronization transition^19–21^. In particular, Franović et al. revealed that when there are interaction delays among neurons and they are influenced by external noise interactions, as well as with the excitability of individual neuron, synchronization clusters spontaneously emerge in the system^22^. Furthermore, time delay has been proved to play a crucial role in inducing SR in neural networks. For instance, Wang et al. discovered that time delay can induce the occurrence of SMR at every multiple of the pacemaker period in scale-free neural networks, facilitated by the presence of moderate noise^23^. Similarly, Liu et al. showed that when time delay is appropriately tuned in feed-forward-loop neuronal network motifs, multiple- SRs can appear^24^. Sun et al. reported that, regardless of whether the noise intensity was optimal or not, the detection capability of signals can be enhanced through the introduction of time delay in excitable neural networks^25^. Yu et al. revealed that the interaction between hybrid electrochemical synapses and time delay enhances the efficiency of signal transmission and promotes the detection capability of subthreshold signals, thereby strengthening the phenomenon of SR^26^. Recently, Tuo et al. demonstrated that the strength of delay-induced SMR can be controlled by adjusting the learning rate of synaptic plasticity in modular neural networks^27^. Yu et al. found that time delay can cause period variations in the signal transmission, resulting in SMR occurring at integer multiples of half the signal period in excitable neural networks^28^. Furthermore, they revealed that in delay-coupled neural networks, weak coupling can enhance the performance of signal responses within a broader range of delay windows, thus promoting the emergence of SMR induced by time delay^29^. Therefore, the investigation of the influence of time delay on SR in neural networks continues to be a prominent and popular research topic.

It is widely acknowledged that there are several traditional indicators, such as the signal-to-noise ratio (SNR)^30^, spectral power amplification^31^, Fourier coefficients^32^, and others^33^, which are employed to measure SR in neural networks. These indicators assist in the detection of weak signals and enhance our understanding of information transmission. However, there are limited studies focusing on SR in neural networks from the perspective of information theory, using SCM as a metric. Indeed, SCM is also an important quantifier to characterize SR, which has been profoundly investigated in low dimensional systems^34–36^. It has been illustrated that SCM could describe subtle features of internal systems. In a notable study, Sun et al. successfully showcased the efficacy of SCM in detecting double-SRs in fractional-order bistable systems, encompassing both strong and weak resonances^37^. In contrast, SNR can only capture the presence of strong resonances. This highlights that SCM is more effective and accurate than SNR in detecting weak signals within low-dimensional bistable systems. Building upon this, Wu et al. further expanded the application of SCM to measure SR behaviors in neural networks, validating its effectiveness^38^. However, their investigation did not consider the influence of time delay on SR in neural networks.

Inspired by the findings mentioned above, in order to reveal more subtle firing dynamical characteristics and delve deeper into the impact of time delay on weak signals detection in neural networks, it becomes crucial to investigate delay-induced SR using SCM as a tool in this study. Accordingly, the remainder of this paper is organized as follows: "Model and methodology" presents the mathematical model and introduces the utilization of SCM. Main results are exhibited in "Results". Finally, some conclusions are drawn in "Conclusion".

## Model and methodology

### Mathematical model

The considered neural network is the ensemble of FHN neurons^39,40^, governed by the following equations

1 $$\begin{gathered} \varepsilon \dot{x}_{i} = x_{i} - \frac{{x_{i}^{3} }}{3} - y_{i} + g\sum\limits_{j = 1}^{N} {J_{ij} } \left( {x_{j} \left( {t - \tau } \right) - x_{i} \left( t \right)} \right), \hfill \\ \dot{y}_{i} = x_{i} + a + A\sin \left( {\frac{2\pi }{{T_{e} }}t} \right) + D\xi_{i} \left( t \right). \hfill \\ \end{gathered}$$

Here $x_{i}$ and $y_{i}$ are separately the fast action potential variable and slow recovery variable of neuron $i$ $\left( {i = 1, \ldots ,N} \right)$, with $N$ being the total number of neurons in the network. $\varepsilon$ represents the small-time scale ratio between the fast and slow variables. By fixing $\varepsilon$ at 0.01 in this study, we are able to accurately describe the rapid evolution of membrane potential in the FHN neural network model and capture the dynamic characteristics of the system. $g$ is the coupling strength. In this work, we consider a Watts-Strogatz (WS) small-world network with $N$ neurons. To obtain it, a regular ring-like network is firstly constructed, where each unit is connected to its $k$ nearest neighbors, then rewiring each edge randomly with the probability $p$. Based on the employed network, if neuron $i$ is connected to neuron $j$, then $J_{ij} = J_{ji} = 1$, otherwise $J_{ij} = J_{ji} = J_{ii} = 0$. $\tau$ is the time delay. The behavior of each individual neuron is determined by the system parameter $a$, where $\left| a \right| < 1$ signifies oscillatory behavior and $\left| a \right| > 1$ indicates excitable behavior. In this study, we assume that all units exhibit excitable behavior by fixing a value of $a = 1.1$ for every unit. $A\sin \left( {\frac{2\pi }{{T_{e} }}t} \right)$ represents a subthreshold signal with an amplitude of $A$ and a period of $T_{e}$. That is, this signal cannot initiate neuronal firing without the presence of accompanying noise. Based on the research findings provided by Li et al.^16^, we set $A = 0.14,T_{e} = 14$ to guarantee that this signal remains subthreshold. $D\xi_{i} \left( t \right)$ is a Gaussian white noise with noise intensity $D$, satisfying $\left\langle {\xi_{i} \left( t \right)} \right\rangle = 0,\left\langle {\xi_{i} \left( t \right)\xi_{j} \left( {t^{\prime}} \right)} \right\rangle = \delta_{ij} \delta \left( {t - t^{\prime}} \right)$.

Explicit Euler algorithm is utilized to solve Eq. (1) numerically, where the time step is taken as 0.001 and the initial values are set as $x_{i} \left( 0 \right) = 0,y_{i} \left( 0 \right) = 0$.

### Statistical complexity measure

To well describe the collective response of neural network, we introduce the mean field of membrane potential, denoted as $X\left( t \right) = \frac{1}{N}\sum\nolimits_{i = 1}^{N} {x_{i} } \left( t \right)$, as the output response. Inter-spike interval (ISI) for time series $X\left( t \right)$ is defined as the time interval between two contiguous firings of $X\left( t \right)$, which is recorded as mean ISI (MISI). The structural feature of MISI series is closely related to the generation of SR behavior. Here, we calculate SCM by extracting MISI time series $\left\{ {T_{s} :s = 1, \ldots ,L} \right\}$. To estimate the probability distribution from the time series of MISI, we utilize the Bandt-Pompe (BP) methodology^41^. For a given time series $\left\{ {T_{s} :s = 1, \ldots ,L} \right\}$ and an embedding dimension $d$, we select $d$ elements $\left( s \right) \mapsto = \left( {T_{{s - \left( {d - 1} \right)}} ,T_{{s - \left( {d - 2} \right)}} , \ldots ,T_{s} } \right)$ at times $\left\{ {s,s - 1, \ldots .,s - \left( {d - 1} \right)} \right\}$ with $s$ being arbitrary time. Then an ordered permutation can be obtained by rearranging the $d$ elements, which is expressed as

2 $$T_{{s - r_{{\left( {d - 1} \right)}} }} \le T_{{s - r_{{\left( {d - 2} \right)}} }} \le \cdots \le T_{{s - r_{1} }} \le T_{{s - r_{0} }} ,$$

where $\pi = \left( {r_{0} ,r_{1} , \ldots ,r_{{\left( {d - 1} \right)}} } \right)$ denotes the permutation of $\left( {0,1, \ldots ,d - 1} \right)$. Therefore, the probability distribution $P = \left\{ {p\left( \pi \right)} \right\}$ of the corresponding ordered permutation for $\left\{ {T_{s} :s = 1, \ldots ,L} \right\}$ can be written as

3 $$p\left( \pi \right) = \frac{{\# \left\{ {s\left| {s \le L - d + 1; \, \left( s \right){\text{ has type }}{\kern 1pt} {\kern 1pt} {\kern 1pt} \pi } \right.} \right\}}}{L - d + 1},$$

with $\#$ being the number of corresponding permutations.

For an example, setting $\left\{ {T_{s} } \right\} = \left\{ {1.1,3.5,2.3,4.7,1.8,5.6} \right\}$ and the embedding dimension $d = 3$, we can obtain $\left\{ {1.1,3.5,2.3} \right\} \mapsto \left( {0,2,1} \right)$, $\left\{ {3.5,2.3,4.7} \right\} \mapsto \left( {1,0,2} \right)$, $\left\{ {2.3,4.7,1.8} \right\} \mapsto \left( {2,0,1} \right)$, $\left\{ {4.7,1.8,5.6} \right\} \mapsto \left( {1,0,2} \right)$ by comparing three adjacent elements. For instance, given the vector $\left\{ {\gamma_{0} ,\gamma_{1} ,\gamma_{2} } \right\} = \left\{ {3.5,2.3,4.7} \right\}$, we achieve $\gamma_{1} < \gamma_{0} < \gamma_{2}$, which leads to the permutation $\left( {1,0,2} \right)$. Therefore, we can acquire the probabilities for each permutation as follows: $p\left[ {\left( {0,1,2} \right)} \right] = 0$, $p\left[ {\left( {0,2,1} \right)} \right] = {1 \mathord{\left/ {\vphantom {1 4}} \right. \kern-0pt} 4}$,$p\left[ {\left( {1,0,2} \right)} \right] = {2 \mathord{\left/ {\vphantom {2 4}} \right. \kern-0pt} 4}$, $p\left[ {\left( {1,2,0} \right)} \right] = 0$, $p\left[ {\left( {2,0,1} \right)} \right] = {1 \mathord{\left/ {\vphantom {1 4}} \right. \kern-0pt} 4}$, $p\left[ {\left( {2,1,0} \right)} \right] = 0$.

Next, we use $P = \left\{ {p_{i} ,i = 1, \ldots ,M} \right\}$ to represent the probability distribution of time series $\left\{ {T_{s} :s = 1, \ldots ,L} \right\}$. SCM can be regarded as a function of the probability distribution of MISI time series, expressed as

4 $${\mathcal{C}}[P] = {\mathcal{H}}_{S} [P] \cdot {\mathcal{Q}}_{J} [P,P_{e} ].$$

Here, ${\mathcal{H}}_{S} [P]$ denotes the normalized Shannon-entropy (NSE) with the definition of

5 $${\mathcal{H}}_{S} [P] = {{S[P]} \mathord{\left/ {\vphantom {{S[P]} {S_{\max } }}} \right. \kern-0pt} {S_{\max } }},$$

where $S\left[ P \right] = - \sum\nolimits_{i = 1}^{M} {p_{i} } \ln p_{i}$, representing Shannon's logarithmic information measure, and $S_{\max } = S\left[ {P_{e} } \right] = \ln M$, $P_{e} = \left\{ {{1 \mathord{\left/ {\vphantom {1 {M, \ldots ,{1 \mathord{\left/ {\vphantom {1 M}} \right. \kern-0pt} M}}}} \right. \kern-0pt} {M, \ldots ,{1 \mathord{\left/ {\vphantom {1 M}} \right. \kern-0pt} M}}}} \right\}$. NSE offers a normalized approach to quantify the equilibrium and uncertainty of information, facilitating comparability between various probability distributions. A higher value of NSE indicates a greater degree of uncertainty and disorder in the information, while a lower value implies higher predictability and certainty.

In addition, ${\mathcal{Q}}_{J} \left[ {P,P_{e} } \right]$ is the disequilibrium, quantifying the distance between the current state and the equilibrium state, which is defined as

6 $${\mathcal{Q}}_{J} [P,P_{e} ] = Q_{0} \cdot {\mathcal{J}}[P,P_{e} ].$$

Here $Q_{0}$ is a normalization constant satisfied

7 $$Q_{0} = - 2\left\{ {\left( {\frac{M + 1}{M}} \right)\ln \left( {M + 1} \right) - 2\ln \left( {2M} \right) + \ln M} \right\}^{ - 1} ,$$

and ${\mathcal{J}}[P,P_{e} ]$ represents Jensen-Shannon divergence, determined by

8 $${\mathcal{J}}[P,P_{e} ] = \left\{ {S\left[ {{{\left( {P + P_{e} } \right)} \mathord{\left/ {\vphantom {{\left( {P + P_{e} } \right)} 2}} \right. \kern-0pt} 2}} \right] - {{S\left[ P \right]} \mathord{\left/ {\vphantom {{S\left[ P \right]} 2}} \right. \kern-0pt} 2} - {{S\left[ {P_{e} } \right]} \mathord{\left/ {\vphantom {{S\left[ {P_{e} } \right]} 2}} \right. \kern-0pt} 2}} \right\}.$$

## Results

### Quantifying noise-induced SMR behaviors by SCM

**Figure 1:**
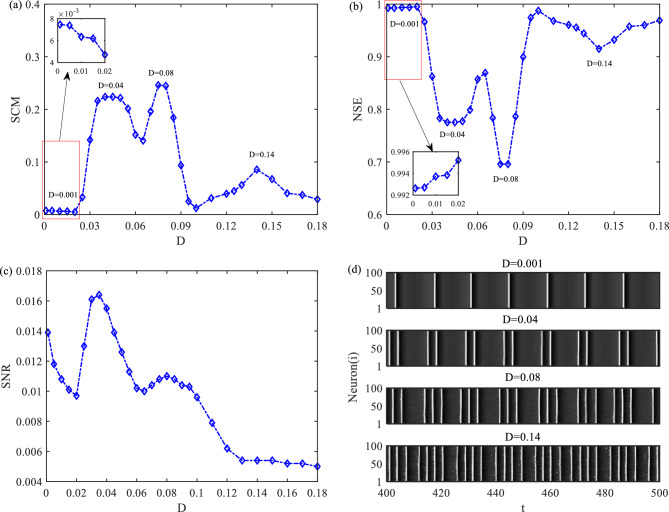
Dependence of SCM, NSE and SNR with respect to noise intensity is shown in (**a–c**), respectively; (**d**) space–time plots with noise intensity $D = 0.001$, $D = 0.04$, $D = 0.08$ and $D = 0.14$.

Here, we keep the model parameters $\varepsilon = 0.01$, $a = 1.1$, $A = 0.14$, $T_{e} = 14$ constant, while setting the network parameters as follows: $N = 100$, $k = 30$, $p = 0.15$, and the coupling strength $g = 0.01$. Then, we will present a discussion on the noise-induced SMR via SCM in model (1) without time delay. We will begin by extracting the MISI time series and estimating their probability distributions using the BP algorithm. Considering the requirements of the BP algorithm, it is necessary to satisfy two conditions: 3 ≤ *d* ≤ 7 for the embedding dimension and *L* >> *d*! for the length of the MISI time series. To ensure a more accurate evaluation of the probability distributions on the MISI time series, we set the embedding dimension to $d = 3$ and the length of the MISI time series to $L = 60,000$. To acquire 60,000 data points, we need to iterate through numerous output response sequences. With these settings, SCM and NSE can be obtained by Eqs. (4) and (5). The results are shown in Fig. 1a, b. Through careful observation, one can read that there are distinct three different noise intensities $D = 0.04$, $D = 0.08$ and $D = 0.14$, around which SCM exhibits prominent maxima, and NSE displays evident minima correspondingly. In addition, there are extra local maximum and minimum on the curves of SCM and NSE at $D = 0.001$. The low value of SCM at $D = 0.001$ can be attributed the distribution of the MISIs sequence approaching a uniform distribution. To highlight the local maximum of SCM and minimum of NSE at $D = 0.001$, partial enlarged drawings of SCM and NSE with respect to noise intensity are respectively plotted in Fig. 1a, b when noise intensity varies in the range of 0.001 to 0.02. Based on these observations, we conclude that SCM achieves four maxima at $D = 0.001$, $D = 0.04$, $D = 0.08$ as well as $D = 0.14$, exhibiting the striking feature of quadruple-SRs.

To verify, we plot the dependence of SNR with respect to noise intensity in Fig. 1c and present the spatiotemporal patterns of neuronal firing activities for $D = 0.001$, $D = 0.04$, $D = 0.08$ and $D = 0.14$ in Fig. 1d. In this analysis, we adopt the common definition of SNR, represented as $SNR = {{P_{S} } \mathord{\left/ {\vphantom {{P_{S} } {P_{N} }}} \right. \kern-0pt} {P_{N} }}$, where $P_{S}$ is the power of the signal and $P_{N}$ is the power of the noise. To obtain the results for SNR, we conducted 30 numerical simulations. From Fig. 1c, it is evident that the SNR curve exhibits three maxima at nearly the same three optimal noise intensities as the SCM curve. However, SNR does not reach its maximum at $D = 0.14$, which is different from SCM. Furthermore, Fig. 1d reveals that firing activities of neurons not only display high periodicity at $D = 0.001$, $D = 0.04$, $D = 0.08$, but also show strong regularity at $D = 0.14$, with periods approximately equal to that of the subthreshold signal. Therefore, the subthreshold signal can be detected efficiently, and both SCM and NSE attain their respective maxima and minima at these four noise intensities. We can conclude that, compared with the traditional SNR indicators, SCM and NSE indicators are more easily to detect weak periodic signals when noise intensity is strong. Thus, SCM may be a better candidate to quantify SMR behaviors and further detect the weak periodic signals in coupled excitable FHN models. Remarkably, the firing patterns of neurons exhibit noticeable differences under these four noise intensities. When $D = 0.001$, neurons generate one spike in a period, while at $D = 0.04$, $D = 0.08$ and $D = 0.14$, neurons separately generate two, three and four spikes in a period. To denote these distinct firing activities, we refer to them as period-1, period-2, period-3, and period-4 firing activities, respectively.

Based on the analysis results from Li et al*.*^16^, we can conclude that, on one hand, the occurrence of noise-induced quadruple-SRs can be attributed to the interaction between the internal time scale induced by the noise *T*_0_ and the period of the subthreshold signal $T_{e}$ in the neuronal network. *T*_0_ denotes the firing period of neuronal activities in the absence of external signals, corresponding to the emergence of CR in the neural network. On other hand, if the period of subthreshold signal $T_{e}$ falls within the range of $\left( {m \times T_{0} ,\left( {m + 1} \right) \times T_{0} } \right)$, noise has the capability to induce SR for $m$ times with $m$ being a positive integer. Following the method described by Li et al.^16^, we have determined that *T*_0_ is approximately 3.45 for our calculations. Therefore, with $T_{e}$ set at 14, noise can induce four occurrences of SR in our neural network model.

### Detecting delay-induced SMR behaviors by SCM

In this subsection, when keeping the model parameters $\varepsilon = 0.01$, $a = 1.1$, $A = 0.14$,$T_{e} = 14$, $g = 0.01$ fixed, and the network parameters $N = 100$, $k = 30$, $p = 0.15$ unchanged, we mainly use SCM to investigate SMR induced by time delay with the help of noise, and further to gain more insight into the effect of time delay on detecting the subthreshold signal in small-world neural networks. Here the embedding dimension and length of MISI time series for different time delays are always fixed as $d = 3$ and $L = 60000$. Besides, we propose a hypothesis that if neurons do not generate firing spike under certain values of time delay, SCM and NSE are treated as 0 and 1, respectively. Based on the results of noise-induced SMR in the absence of time delay, we will pay attention to study effects of time delay on detecting the subthreshold signal under these noise intensities of $D = 0.02$, 0.04, 0.065, 0.08 and 0.14, respectively.

**Figure 2:**
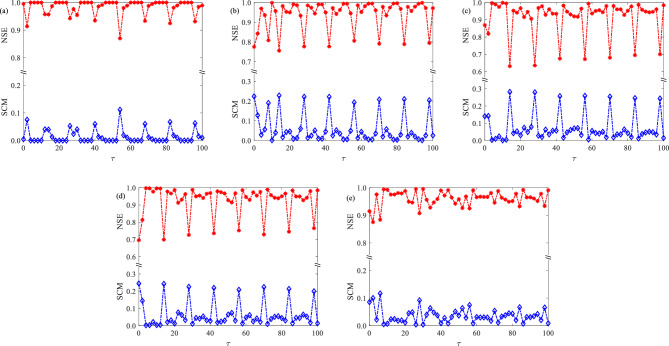
Dependence of SCM and NSE on different noise intensities with respect to time delay, where (**a**) $D = 0.02$, (**b**) $D = 0.04$, (**c**) $D = 0.065$, (**d**) $D = 0.08$ and (**e**) $D = 0.14$.

**Figure 3:**
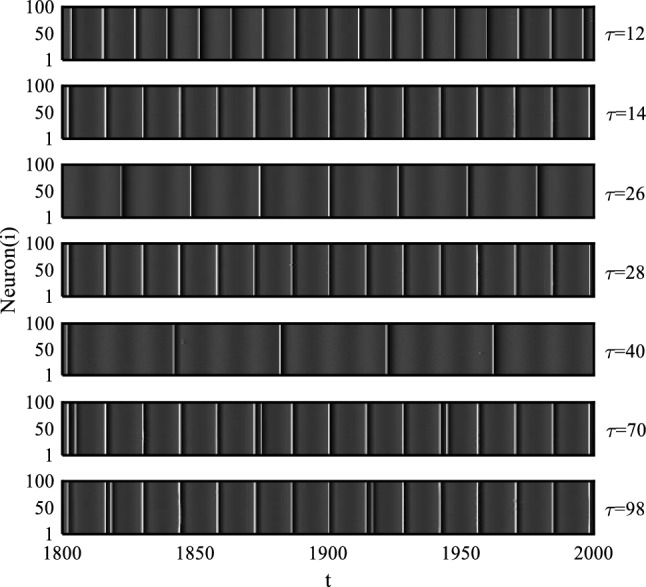
Space–time plots with different values of time delay under $D = 0.02$.

Dependence of SCM and NSE on time delay with $D = 0.02$, 0.04, 0.065, 0.08 and 0.14 is shown in Fig. 2. From Fig. 2a, it is evident that for $D = 0.02$, the SCM curve exhibits a series of local maxima at approximately $\tau { = }2$, 12, 26, 40, 54, 68, 82, and 96, while also achieving large values at $\tau { = }14$ and 28. Conversely, the NSE curve displays an opposite trend. With the results presented in Fig. 3, we observe that neurons exhibit regular firing activities at $\tau { = }12$, 26, and 40, where their firing periods align appropriately with the values of time delay. This observation suggests a strong coherence between time delay and neuronal firing dynamics at these specific time delay values. However, at $\tau { = }14$ and 28, neuronal firing activities demonstrate periodicity with their periods closely resembling the period of the subthreshold signal. This finding establishes a significant correlation between the subthreshold signal and neuronal firing dynamics at these two values of time delay, leading to the occurrence of SMR. Notably, we can see that SCM does not achieve optimal values at $\tau = 56,70,84$ and 98. Additionally, as evidenced by the results in Fig. 3, the neuronal firing activities at $\tau = 70$ and 98 are non-periodic, further confirming that the subthreshold signal cannot be detected successfully and SMR does not occur in these cases. Therefore, it is summarized that under a low noise level, time delay can induce the emergence of multiple-CRs when the time delay is small or fixed around values of ${\text{n}}T_{e} - 2$, with ${\text{n}}$ being a positive integer. Additionally, time delay can give rise to SMR occurring at ${\text{n}}T_{e}$, where ${\text{n}}$ is a small positive integer under low noise conditions. In particular, it is worth mentioning that at $\tau = 26$ and 40, the values of SCM are slightly larger than at $\tau = 28$, suggesting that in the case of $D = 0.02$, the strength of CR is quite greater than that of SR. How can we distinguish between SR and CR by observing the curve of SCM with respect to time delay? We can identify this based on the correlation between time delay and the period of subthreshold signal. If SCM reaches a relatively large value at an integer multiple of the period of subthreshold signal, it is considered as exhibiting SR induced by time delay in the system. On the other hand, if a local maximum is achieved at time delay deviating from an integer multiple of the period of subthreshold signal, it is believed to experience delay-induced CR in the system.

Surprisingly, the investigation reveals that with an increase of noise intensity, such as $D = 0.04$, 0.065, and 0.08, as presented in Fig. 2b–d, several noticeable local maxima of SCM and corresponding pronounced local minima of NSE occur at $\tau { = }14$, 28, 42, 56, 70, 84, and 98. These observations differ from the findings depicted in Fig. 2a. These outcomes, illustrated in Fig. 2b–d, indicate that delay-induced SMR phenomena can manifest at $\tau {\text{ = n}}T_{e}$, where ${\text{n}}$ represents a positive integer. This implies that the detection ability of subthreshold signal is enhanced when the values of time delay equal to multiples of the period of subthreshold signal, especially under moderate levels of noise. Furthermore, it is worth noting that under these noise levels, other prominent maxima may also appear on the SCM curves when time delay is small. Taking $D = 0.04$ and 0.065 as examples, it can be observed that optimal values of time delay, namely 8 and 2, respectively, lead to local maxima on the SCM curves. This phenomenon can be attributed to the strong correlation between time delay and neuronal firing dynamics. Interestingly, when noise intensity becomes very high, as shown in Fig. 2e for $D =0.14$, both SCM and NSE trajectories become chaotic with increasing time delay. Combining the findings presented in Fig. 2b–e, it can be concluded that under high levels of noise, the delay-induced SMR phenomena are decreased, resulting in a weakened ability to detect the subthreshold signal. Moreover, for these four noise intensities, between the optimal values of time delay, multiple small peaks can be observed on the SCM curves, suggesting the existence of multiple resonance-like phenomena between delay-induced SMR. Importantly, we have deduced that in the absence of time delay, the subthreshold signal cannot be efficiently detected at $D = 0.02$ and 0.065 due to the presence of local minima on SCM curves, as depicted in Fig. 1a. However, when time delay is taken into account in the information transmission between neurons, the detection capability of subthreshold signal is significantly enhanced under these two noise intensities, as demonstrated in Fig. 2c, d. Thus, we can conclude that time delay can effectively assist noise in detecting the subthreshold signal.

**Figure 4:**
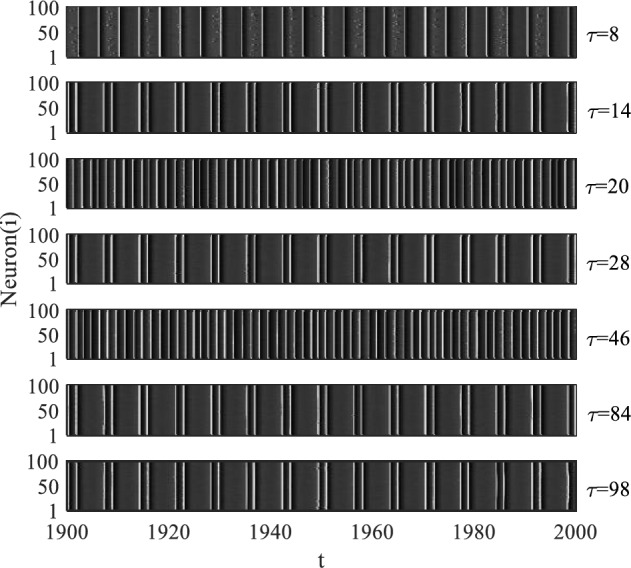
Space–time plots with different values of time delay under $D = 0.04$.

To further investigate the role of time delay in signal detection, we present the spatiotemporal patterns of neuronal firing activities for different time delays under $D = 0.04$, as depicted in Fig. 4. The figure indicates that neurons exhibit approximately periodic firing activities at various time delays, including $\tau = 8$, 14, 20, 28, 46, 84, and 98. Notably, firing activities at $\tau = 8$, 14, 28, 46, 84, and 98 display more pronounced periodicity compared to other time delays such as $\tau = 20$ and 46. These significant periodicities are accompanied by large local maxima on the SCM curves at $\tau = 8$, 14, 28, 46, 84, and 98, as observed in Fig. 2b. Additionally, when $\tau {\text{ = n}}T_{e}$, with ${\text{n}}$ being a positive integer (e.g., $\tau = 14$, 28, 84, and 98), the firing dynamics of neurons resemble those without time delay. The periods of firing activities during these time delays are nearly identical to the period of subthreshold signal. This implies that, under $D = 0.04$, the subthreshold signal can be efficiently detected and the delay-induced SMR phenomena occur at $\tau {\text{ = n}}T_{e}$, with ${\text{n}}$ being a positive integer. However, for $\tau = 8$, 20, and 46, the periods of firing activities deviate from the period of the subthreshold signal, leading to a failure in detecting the subthreshold signal. Particularly, the period of firing activities at $\tau = 8$ is almost equal to the value of time delay, indicating a strong coherence between time delay and neuronal firing dynamics. In conclusion, it can be inferred that when time delay is equal to multiples of the period of the subthreshold signal, time delay can enhance the detection ability of subthreshold signal under moderate noise levels, resulting in the delay-induced SMR phenomena.

### Mechanism of delay-induced SMR

In this subsection, we will explain the phenomena of time delay-induced SMR. The results shown in Fig. 2 demonstrate that, under appropriate noise intensities, the SMR behaviors induced by time delay can occur at the integer multiples of the period of subthreshold signal. We believe that these phenomena can be attributed to the coupling effect among the time delay, the subthreshold signal and the firing dynamics of neurons. In detail, when time delay aligns with integer multiples of the subthreshold signal's period, the coupling among these factors leads to mutual activation among neurons, enhancing signal propagation efficiency and guiding neuronal firing dynamics to the optimal response state as delay-free case. As a result, neurons will exhibit synchronized firing patterns that align with the subthreshold signal’s period. This synchronization greatly enhances the sensitivity of the neural network to the subthreshold signal, ultimately resulting in the occurrence of delay-induced SMR.

For example, under the noise intensity of $D = 0.04$, the findings depicted in Fig. 4 reveal that neurons display synchronized and periodic firing activities when the time delay matches integer multiples of the subthreshold signal period. Furthermore, as shown in Fig. 1d, these periodic firing activities are consistent with the firing activities of neurons without time delay. Despite generating two firing activities within one period, the period of neuronal firing patterns equals the period of subthreshold signal, indicating a strong correlation between neuronal firing dynamics and the subthreshold signal during these instances. Consequently, the neural network achieves maximum perceptual sensitivity to the subthreshold signal, resulting in the emergence of multiple resonant responses at integer multiples of the subthreshold signal period. However, when the values of time delay take 8, 20, and 46, the firing patterns of neurons, although relatively regular, do not match the period of subthreshold signal, indicating no correlation between neuronal firing dynamics and the subthreshold signal. This phenomenon is likely due to the coupling effect between time delay and neuronal firing dynamics, which alters the firing pattern of neurons and gives rise to a coherent dynamic of neuronal firing. Therefore, on the SCM curves that vary with the time delay, a series of prominent peaks occur when the values of time delay equal integer multiples of the subthreshold signal's period, while smaller peaks appear at some specific values of time delay.

### Effects of network parameters on delay-induced SMR

**Figure 5:**
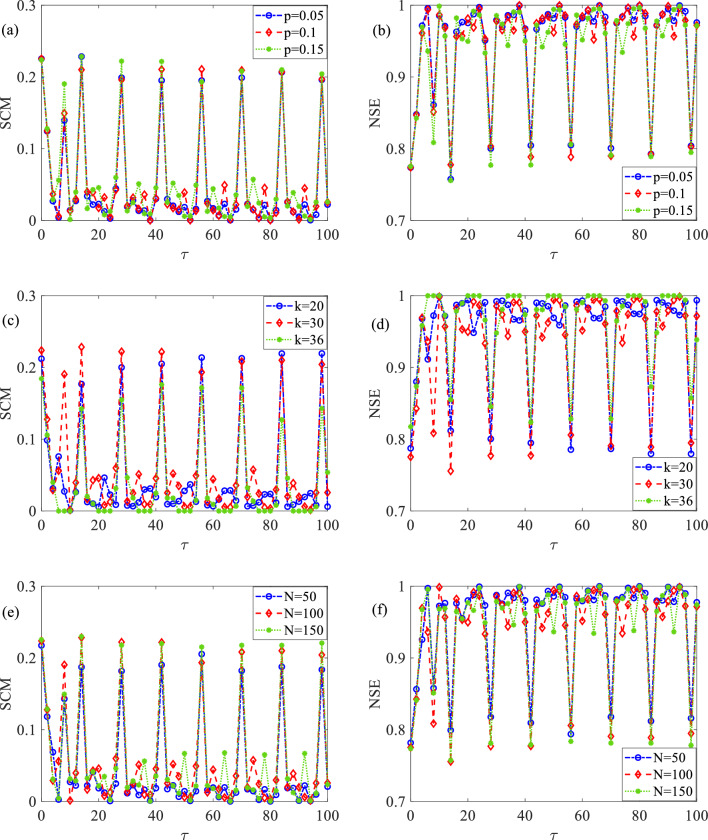
Dependence of SCM and NSE on different network parameters with respect to time delay, where (**a,b**) $k = 30$,$N = 100$ (**c,d**) $p = 0.15$, $N = 100$ (**e,f**) $p = 0.15$, $k = 30$.

Next, we will examine the effects of various network parameters on the occurrence of time delay-induced SMR. Specifically, we focus on the rewiring probability $p$, degree $k$, and network size $N$, while keeping other model parameters $\varepsilon = 0.01$, $a = 1.1$, $A = 0.14$, $T_{e} = 14$ and $g = 0.01$ fixed. The findings are presented in Fig. 5. Analysis of Fig. 5a, b reveals that when $k$ is fixed at 30 and $N$ is set at 100, SCM still exhibits several maxima at $\tau {\text{ = n}}T_{e}$ for different values of $p$, whereas NSE displays an inverse pattern. It is noteworthy that the magnitudes of each peak on the SCM curve and each valley on the NSE curve do not vary significantly. This suggests that the occurrence of delay-induced SMR is robust to the rewiring probability $p$, and the detection ability of subthreshold signal is minimally affected by variations in the rewiring probability.

Similarly, when $p$ is held constant at 0.15 and $N$ remains at 100, Fig. 5c, d demonstrate that a series of maxima of SCM always occur at $\tau {\text{ = n}}T_{e}$, with the increase of $k$, while the opposite pattern is presented on the NSE curve. This indicates the robustness of delay-induced SMR against the degree $k$. However, it is evident that when $\tau < 50$, the height of each peak on the SCM curve first increases and then decreases with the increase of $k$. This implies the existence of an optimal value of $k$ that facilitates the enhancement of delay-induced SMR under moderate time delays. But, when $\tau > 50$, the height of every peak of SCM decreases as $k$ increases, suggesting that higher degrees of small-world networks may weaken the strength of delay-induced SMR for large time delays. Consequently, by adjusting the degrees of small-world networks, we can effectively control the phenomena of delay-induced SMR and maximize the detectability of subthreshold signal.

Moreover, when $p$ remains at 0.15 and $k$ stays constant at 30, SCM and NSE display a series of maxima and minima at $\tau {\text{ = n}}T_{e}$ for various values of $N$, as illustrated in Fig. 5e, f. This highlights the robustness of delay-induced SMR in relation to the network size $N$. Upon careful examination, it is evident that larger network sizes result in higher peak heights for SCM and lower valley depths for NSE. This suggests that larger network sizes effectively strengthen delay-induced SMR. Therefore, to promote the detection efficiency of subthreshold signal, it is advantageous to appropriately expand the network sizes of small-world networks.

### Effect of coupling strength on delay-induced SMR

**Figure 6:**
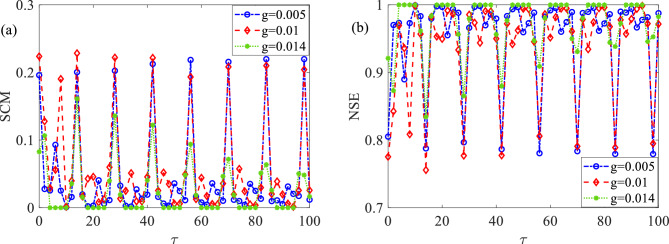
Dependence of SCM and NSE on different coupling strengths with respect to time delay.

When keeping the model parameters $\varepsilon = 0.01$, $a = 1.1$, $A = 0.14$, $T_{e} = 14$ constant, and the network parameters $N = 100$, $k = 30$, $p = 0.15$ unchanged, we explore the impact of coupling strength $g$ on delay-induced SMR, as displayed in Fig. 6. From the figure, it can be observed that for $\tau < 50$, the peaks of SCM initially increase and then decrease with the rising $g$, while NSE shows the opposite trend. This suggests the presence of an optimal coupling strength that maximizes the intensity of delay-induced SMR within suitable delay values. However, for $\tau > 50$, the peaks of SCM decrease while the valleys of NSE increase as $g$ increases. This indicates that weaker coupling strengths are more advantageous for enhancing the effect of delay-induced SMR when dealing with larger delay values. Hence, by adjusting the coupling strength, we can optimize the occurrence of delay-induced SMR and maximize the detection capability of subthreshold signal.

## Conclusion

The current research is devoted to detecting and quantifying delay-induced SMR by virtue of SCM in small-world neural networks, which are locally modeled by FHN neurons. MISI time series have been firstly extracted and BP algorithm has been employed to evaluate their probability distributions, based on which SCM and NSE are defined and calculated numerically. Without delay, there are four maxima of SCM and corresponding four minima of NSE at four optimal noise levels, indicating the generation of SMR phenomena induced by noise. SNR has been also carried out to characterize the noise-induced SMR. It has been found that SCM may be a better quantifier to detect SMR in neural networks than SNR. However, when time delay is introduced into the coupling process, SR can be either enhanced or decreased by time delay in small-world neural networks. At a low noise level, an intriguing phenomenon occurs: an increasing time delay leads to the emergence of regular spatial–temporal orders in neuronal firing dynamics. Furthermore, it is observed that the periods of regular firing activities in neurons align closely with the optimal values of time delay, which indicates the strong coherence between time delay and neuronal firing dynamics. Indeed, the firing periods of neurons will undergo variations in response to changes in time delay. In the presence of moderate noise intensity, SMR phenomena can occur at integer multiples of the subthreshold signal’s period, which are characterized by some prominent local maxima of SCM. Under high noise intensity conditions, SCM exhibits irregularity with varying time delay, leading to the weakness of delay-induced SMR. Finally, we have revealed the robustness of network parameters and coupling strength in the occurrence of delay-induced SMR, and demonstrated that the degrees and sizes of small-world networks, as well as the coupling strength can regulate the strength of delay-induced SMR.

It is well known that weak signals are ubiquitous in brain and usually encode the neural information. We thus expect that SCM, as an effective tool, can be used in weak signals detection in neural systems. There remains one lingering problem on how to evaluate the optimal values for subthreshold signals that are masked by noise. Perhaps we can refer to the study by Liang et al.^42^, where they controlled parameter diversity and coupling strength to jointly modulate the waveform or period of collective activity in the system, resulting in resonance for optimal suprathreshold signals. This subject holds significant importance for applications in the field of signal processing.

## Data Availability

The datasets used and/or analysed during the current study available from the corresponding author on reasonable request.

## References

[1] Benzi R, Sutera A, Vulpiani A (1981). The mechanism of stochastic resonance. J. Phys. A Math. Gen..

[2] Xu PF, Jin YF (2020). Stochastic resonance in an asymmetric tristable system driven by correlated noises. Appl. Math. Model..

[3] Hänggi P (2002). Stochastic resonance in biology how noise can enhance detection of weak signals and help improve biological information processing. ChemPhysChem.

[4] Lu SL, He QB, Wang J (2019). A review of stochastic resonance in rotating machine fault detection. Mech. Syst. Signal. Pr..

[5] Shi ZZ, Liao ZQ, Tabata H (2022). Boosting learning ability of overdamped bistable stochastic resonance system based physical reservoir computing model by time-delayed feedback. Chaos Soliton. Fract..

[6] Douglass JK, Wilkens L, Pantazelou E, Moss F (1993). Noise enhancement of information transfer in crayfish mechanoreceptors by stochastic resonance. Nature.

[7] Levin JE, Miller JP (1996). Broadband neural encoding in the cricket cereal sensory system enhanced by stochastic resonance. Nature.

[8] Russell DF, Wilkens LA, Moss F (1999). Use of behavioural stochastic resonance by paddle fish for feeding. Nature.

[9] Gluckman BJ, Netoff TI, Neel EJ, Ditto WL, Spano ML, Schiff SJ (1996). Stochastic resonance in a neuronal network from mammalian brain. Phys. Rev. Lett..

[10] Mori T, Kai S (2002). Noise-induced entrainment and stochastic resonance in human brain waves. Phys. Rev. Lett..

[11] Leng G, Brown CH, Russell JA (1999). Physiological pathways regulating the activity of magnocellular neurosecretory cells. Prog. Neurobiol..

[12] Moss F, Ward LM, Sannita WG (2004). Stochastic resonance and sensory information processing: A tutorial and review of application. Clin. Neurophysiol..

[13] Perc M, Gosak M (2008). Pacemaker-driven stochastic resonance on diffusive and complex networks of bistable oscillators. New J. Phys..

[14] Kwon O, Jo HH, Moon HT (2005). Effect of spatially correlated noise on coherence resonance in a network of excitable cells. Phys. Rev. E.

[15] Liu HX, Lu LL, Zhu Y, Wei ZC, Yi M (2022). Stochastic resonance: The response to envelope modulation signal for neural networks with different topologies. Physica A.

[16] Li HX, Sun XJ, Xiao JH (2018). Stochastic multiresonance in coupled excitable FHN neurons. Chaos.

[17] Liang XM, Tang M, Dhamala M, Liu ZH (2020). Phase synchronization of inhibitory bursting neurons induced by distributed time delays in chemical coupling. Phys. Rev. E.

[18] Roxin A, Brunel N, Hansel D (2005). Role of delays in shaping spatiotemporal dynamics of neuronal activity in large networks. Phys. Rev. Lett..

[19] Ma J, Tang J (2017). A review for dynamics in neuron and neuronal network. Nonlinear Dyn..

[20] Shafiei M (2019). Effects of partial time delays on synchronization patterns in Izhikevich neuronal networks. Eur. Phys. J. B.

[21] Wang QY, Duan ZS, Perc M, Chen GR (2008). Synchronization transitions on small-world neuronal networks: Effects of information transmission delay and rewiring probability. EPL.

[22] Franović I, Todorović K, Vasović N, Burić N (2012). Spontaneous formation of synchronization clusters in homogenous neuronal ensembles induced by noise and interaction delays. Phys. Rev. Lett..

[23] Wang QY, Perc M, Duan ZS, Chen GR (2009). Delay-induced multiple stochastic resonances on scale-free neuronal networks. Chaos.

[24] Liu C, Wang J, Yu H, Deng B, Tsang KM, Chan WL, Wong YK (2014). The effects of time delay on the stochastic resonance in feed-forward-loop neuronal network motifs. Commun. Nonlinear Sci. Numer. Simulat..

[25] Sun XJ, Liu ZF (2018). Combined effects of time delay and noise on the ability of neuronal network to detect the subthreshold signal. Nonlinear Dyn..

[26] Yu H, Guo X, Wang J (2017). Stochastic resonance enhancement of small-world neural networks by hybrid synapses and time delay. Commun. Nonlinear Sci. Numer. Simulat..

[27] Tuo XH, Yang XL (2022). How synaptic plasticity affects the stochastic resonance in a modular neuronal network. Nonlinear Dyn..

[28] Yu D, Wang GW, Ding QM, Li TY, Jia Y (2022). Effects of bounded noise and time delay on signal transmission in excitable neural networks. Chaos Soliton Fract..

[29] Yu D, Wu Y, Yang L, Zhao Y, Jia Y (2023). Effect of topology on delay-induced multiple resonances in locally driven systems. Physica A.

[30] Semenov VV, Zakharova A (2022). Multiplexing-based control of stochastic resonance. Chaos.

[31] Yang X, Yu Y, Sun Z (2017). Autapse-induced multiple stochastic resonances in a modular neuronal network. Chaos.

[32] Yang H, Xu G, Wang H (2022). Effects of magnetic fields on stochastic resonance in Hodgkin–Huxley neuronal network driven by Gaussian noise and non-Gaussian noise. Cogn. Neurodyn..

[33] Kanamaru T, Okabe Y (2000). Stochastic resonance in a pulse neural network with a propagational time delay. BioSystems.

[34] Rosso OA, Masoller C (2009). Detecting and quantifying temporal correlations in stochastic resonance via information theory measures. Eur. Phys. J. B.

[35] Rosso OA, Masoller C (2009). Detecting and quantifying stochastic and coherence resonances via information-theory complexity measurements. Phys. Rev. E.

[36] He MJ, Xu W, Sun ZK, Jia WT (2017). Characterizing stochastic resonance in coupled bistable system with Poisson white noises via statistical complexity measures. Nonlinear Dyn..

[37] Sun ZK, Dang PN, Xu W (2019). Detecting and measuring stochastic resonance in fractional-order systems via statistical complexity. Chaos Soliton. Fract..

[38] Wu YZ, Sun ZK, Liu YY (2023). Detecting the subthreshold signal in a neural network via statistical complexity measure. Phys. Scr..

[39] FitzHugh R (1961). Impulses and physiological states in theoretical models of nerve membrane. Biophys. J.

[40] Nagumo J, Arimoto S, Yoshizawa S (1962). An active pulse transmission line simulating nerve axon. Proc. IRE.

[41] Bandt C, Pompe B (2002). Permutation entropy: A natural complexity measure for time series. Phys. Rev. Lett..

[42] Liang XM, Zhang XY, Zhao L (2020). Diversity-induced resonance for optimally suprathreshold signals. Chaos.

